# Effect of a standardized maternal meal on fetal middle cerebral artery Doppler indices: A single-blinded crossover study

**DOI:** 10.1371/journal.pone.0272062

**Published:** 2022-08-04

**Authors:** Saba Muneer Zahid, Gun Lisbet Opheim, Tore Henriksen, Trond Melbye Michelsen, Guttorm Haugen

**Affiliations:** 1 Division of Obstetrics and Gynaecology, Department of Fetal Medicine, Oslo University Hospital, Oslo, Norway; 2 Faculty of Medicine, Institute of Clinical Medicine, University of Oslo, Oslo, Norway; 3 Division of Obstetrics and Gynaecology, Department of Obstetrics, Oslo University Hospital, Oslo, Norway; Metrohealth Medical Center, UNITED STATES

## Abstract

**Objective:**

Measures of Doppler blood flow velocity profiles are an integral part of monitoring fetal well-being during pregnancy. These examinations are performed at different times of the day and at different maternal meal states. In uncomplicated pregnancies, we assessed the effect of a standardized maternal meal on middle cerebral artery (MCA) and umbilical artery (UA) Doppler blood flow velocity pulsatility indices (PIs) and MCA peak systolic velocity (PSV).

**Methods:**

In this prospective single-blinded crossover study 25 healthy women were examined at 36 weeks of pregnancy. The first examination was performed in the morning following overnight fast, and repeated after extended fast (state A), and after a standard breakfast meal (state B).

**Results:**

Irrespective of maternal prandial status, the MCA-PI values were lower in the 2^nd^ compared to the 1^st^ examination (-0.187; p = 0.071, and -0.113; p = 0.099, state A and B, respectively). Compared to the values in the 1^st^ examination, the UA-PI values, were higher after extended fast (0.014; p = 0.436), and lower post-prandially (-0.036; p = 0.070). The difference (state B minus state A) between the meal states were not significant (0.074; p = 0.487 and -0.050; p = 0.058, for MCA-PI and UA-PI, respectively). Adjusting for the possible influence of fetal heart rate on MCA-PI and UA-PI, the differences between meal states remained non-significant (p = 0.179, p = 0.064, respectively). The MCA-PSV values increased after the meal (6.812; p = 0.035), whereas no increase was observed following extended fast (0.140; p = 0.951). The difference in MCA-PSV values between the two meal states was not significant (6.672; p = 0.055).

**Conclusion:**

Our results demonstrate possible diurnal variations in MCA-PI and UA-PI, with and without adjustment for fetal heart rate, that seem to be unaffected by maternal meal intake in healthy pregnancies.

## Introduction

Doppler ultrasound measures of the fetal middle cerebral artery (MCA) and umbilical artery (UA) blood flow velocity waveforms, e.g. pulsatility indices (PIs), are an integral part of fetal surveillance. In clinical practice these examinations are performed at differing times of the day, and at different maternal meal states (fasting vs. after meal intake).

Fetal hypoxemia is related to redistribution of the fetal circulation, with elevated blood flow to the cerebrum at the expense of the viscera. This is reflected as reduced resistance to flow in the MCA and increased resistance in the UA [1–3]. A decrease in the cerebroplacental Doppler ratio (CPR; MCA PI divided by UA PI) indicates a redistribution of fetal blood flow to prioritize the brain. A low CPR in both small-for-gestational-age (SGA) and appropriate-for-gestational-age (AGA) fetuses is associated with adverse perinatal and neonatal outcomes [4–7]. Fetal hypoxemia and hypercapnia are also associated with an elevated MCA peak systolic velocity (PSV) in early SGA fetuses (<32 weeks of gestation) [8]. Moreover, an evaluation of increased MCA PSV is used as a noninvasive procedure to detect fetal anemia [9].

In utero, maternal circadian signals (e.g., melatonin, cortisol, temperature) seem to be an important factor for fetal circadian systems and peripheral clocks [10]. The fetal-uteroplacental circulation represents a complex system with maternal, fetal and placental physiological features that can affect fetal Doppler blood flow velocity indices [11–15]. This includes the possible impact of the maternal prandial status on the UA and MCA Doppler values. Previous studies from our group indicated that maternal non-physiological glucose loading in gestational week (GW) 30–32 and maternal meal intake in GW 36 induced a decrease in MCA PI values [16, 17]. Week 36 is towards the end of the energy-depositing phase of pregnancy, during which maternal nutritional intake may be particularly influential [18]. Our research also demonstrated a decrease in MCA PI values adjusted for FHR after a meal. As these studies did not include a control group the influence of maternal diurnal variation on the fetal blood velocities could not be excluded. In the current study we hypothesize that compared to an extended fast (i.e. continuing an overnight fast until after lunch time), a maternal meal will reduce MCA PI, both dependent and independent of FHR, at gestational week 36. The aim of the present study was to assess the effect of a maternal meal on indices of cerebrovascular and fetoplacental vascular resistance in uncomplicated third trimester pregnancies using a blinded, crossover design.

## Material and methods

In this single-blinded crossover study, we invited women attending the routine ultrasound examination between gestational week 18 and 20 to participate in the study. Twenty-seven women of Caucasian origin with singleton pregnancies entered the study from January 2019-May 2020. Reasons for exclusion were pre-existing medical conditions, obstetric complications, food intolerance or food allergy, use of medication with potential risk of affecting fetoplacental and fetal circulation, and fetuses with malformations or chromosomal aberrations. As part of national routine practice in Norway, the gestational age was estimated by ultrasound measurement of head circumference (HC) in GW 18–20 [19].

### Subjects

**Fig 1:**
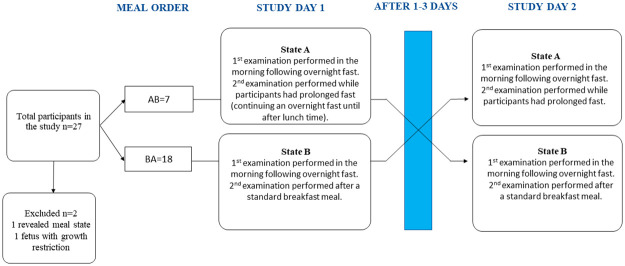
Study design flowchart. Each participant was examined twice: on study day 1 and study day 2 (each separated by 1–3 days). The order of the two meal states A and B, were allocated to participants to either: AB (state A study day 1 and state B study day 2), or BA (state B study day 1 and state A study day 2).

Each participant underwent the crossover study at GW 36 at two different maternal meal states, state A and state B. The interval between the ultrasound examinations in state A and state B was 1–3 days. The midwife at the ultrasound department consulted and allocated the participants into meal state order AB (N = 7) or BA (N = 18), see Fig 1.

The participants were instructed to fast from midnight (00:00 am), and the first examination was performed at approximately 8:30 am (range; 8:20 am—08:50 am). In state A, the participants continued the fast (prolonged fast) until after the second examination. In state B, the second examination was performed following the standard breakfast meal (SBM) with a mean (±SD) of 104 (±9) minutes. The second examination started approximately at 11:15 am (range; 11:00 am—12:30 pm). The time interval from the end of the first examination to the start of the second examination in state A and B were 125 (±10) and 126 (±9) minutes, respectively.

The SBM contained approximately 400 kcal, and consisted of two slices of bread with cheese and ham, one boiled egg, one standard package with jam and one with butter, one glass (about 150 ml) of 1% fat milk and one glass with juice.

One clinician (SMZ) performed all sonographic examinations, and was blinded to the subject allocation process until all data had been collected and analyzed. Each session lasted 36 (±6) minutes. After birth, information on birth weight (BW), length, gender and Apgar score were collected from medical records.

### Measurements

All the sonographic examinations were performed with an Epic 7 ultrasound system (Philips, Seattle, WA, USA), with a 3 MHz curved linear transducer including color Doppler (2,5 MHz) and pulsed Doppler (3 MHz) facilities. The MCA nearest the transducer was visualized (color Doppler) in a transverse view of the fetal head. Doppler velocity waveforms were sampled from the proximal part of the MCA, near its origin from the internal carotid artery [20]. Doppler tracings in the umbilical artery (UA) were sampled in a free-floating segment of the umbilical cord. Insonation angle was kept as low as possible, always < 30°. For each examination, the sonographer evaluated the fetus as being quiet, active or fairly active during the examination period. However, all velocity waveforms for MCA and UA were recorded at a time with absence of marked fetal body and respiratory movements. PI and FHR were calculated from three consecutive uniform waveforms, and the mean value was used for analysis. The intra-observer variation was analyzed as the intra-class correlation coefficient (ICC) based on a single-rating, consistency, two-way mixed effects model [21]. The ICC estimates were 0.97 for MCA PI (n = 12), 0.95 for UA PI (n = 13), and 0.89 for MCA PSV (n = 12).

Changes in MCA PI, UA PI, FHR, CPR, and MCA PSV values (Δ values) were calculated by subtracting the morning fasting values from extended fasting or postprandial values, in state A and B, respectively.

Fetal biometric measurements included HC, femur length (FL) and abdominal circumference (AC). The mean value of three measurements was calculated. Amniotic fluid index (AFI) was measured during all four ultrasound examinations. At birth, all fetuses were confirmed as appropriate for gestational age, within the mean Norwegian standard curve of birth weight (10^th^ -90^th^ percentile) [22].

Fasting venous blood samples were collected in serum separating tubes after ended first examination. The supernatants were removed and stored at -80° C. Glucose was measured by an accredited laboratory (Department of Medical Biochemistry, Oslo University Hospital) using the hexokinase/glucose-6-phosphate dehydrogenase enzymatic in vitro test (Roche, Mannheim, Germany).

### Statistics

The sample size was calculated, based on results of our previous study on the intake of a regular meal in healthy pregnancies, with a reduction in MCA PI of 0.23 with SD of this difference of 0.4 in GW 36 [17]. This gave a number of 25 pregnancies at week 36 to verify or exclude our hypothesis of a change in MCA PI following a meal compared to extended fast with a power of 80% and significance level of 0.05.

The descriptive data are reported as mean ±SD or 95% confidence interval (CI), median and percentiles, or frequency and percentage, as appropriate. We used paired sample t-test (2-tailed) to examine the differences in the measured Doppler values between 2^nd^ and 1^st^ examination in state A and B, to examine the difference in the measured values at 1^st^ examination between the two study visits (baseline values), and to examine the difference in the measured Δ values (2^nd^ examination minus 1^st^ examination) between the two states (Δ A and Δ B-values).

Several previous studies have demonstrated that FHR and Doppler indices are inversely correlated [23–25]. To adjust for the influence of changes in ΔFHR on ΔPI values, we used a linear mixed model with random intercept at subject level to calculate differences in ΔPI-values, adjusted for their respective ΔFHR-values, between state A and B. ΔPI was set as dependent variable. Meal state and ΔFHR were included as fixed effects in the model [26]. For each artery, the correlation between ΔPI and ΔFHR during each state was based on Pearson correlation.

A p-value < 0.05 was considered statistically significant. Data was analyzed using IBM SPSS Statistics for Windows, version 26.0 (IBM Corp., Armonk., N.Y., USA).

### Ethical approval

The study was approved by the Regional Committee of Medical and Health Research Ethics (Sør-Øst B 2018/1034) and was performed in agreement with the standards outlined in the Helsinki Declaration. Before study entry, all participants gave written informed consent, and institutional guidelines were followed during all procedures.

## Results

Of the 27 recruited women, two were excluded: one because of fetal growth restriction (estimated fetal weight 5p, and birth weight 5p), the other because she revealed the meal state for the ultrasound examiner at the second examination (Fig 1). Twenty-five women were included in the analyses; see Table 1 for maternal, fetal and neonatal characteristics.

**Table 1:** Maternal, fetal and neonatal characteristics (n = 25).

Maternal characteristics	Median (10^th^-90^th^ percentile)	n (%)
Maternal age (years)	32 (28–38)	
Prepregnant body mass index (ppBMI)	21.8 (18.4–26.0)	
Higher education (Bachelor/Bachelor+)		25 (100)
Glucose level in fasting state 1^st^ study day (mmol/L)	4.3 (4.0–4.7)	
Glucose level in fasting state 2^nd^ study day (mmol/L)	4.3 (4.0–5.0)	
**Fetal and neonatal characteristics:**		
Gestational age at 1^st^ study (weeks ^+days^)	36^+1^ (35^+4^–37^+1^)	
Gestational age at 2^st^ study (weeks ^+days^)	36^+3^ (35^+5^–37^+2^)	
Gestational age at delivery (weeks ^+days^)	40^+3^ (39^+0^–41^+6^)	
Head circumference (HC), week 36 (mm)	328 (317–336)	
Abdominal circumference (AC), week 36 (mm)	328 (309–347)	
HC/AC-ratio, week 36	1,00 (0.96–1.04)	
Estimated fetal weight (EFW, Combs) week 36 (g) [27]	2960 (2580–3292)	
Birthweight (g)	3598 (3206–4085)	
Placental weight (g)	660 (546–821)	
Nulliparous		12 (48)
Females		15 (60)
AFI, extended fast (mm)	140 (91–212)	
AFI, after SBM (mm)	140 (102–218)	

AFI; amniotic fluid index, SBM; standard breakfast meal.

Blood samples were obtained in 24 women. There was no significant difference between fasting glucose levels following the first ultrasound examination in study day 1 and study day 2 (-0.05 mmol/L; 95% CI: (-0.20–0.09); p = 0.434). No significant differences in fetal body movements or AFI dependent on prandial state were detected (see Table 1).

### MCA and UA PI, without adjustment for FHR

We obtained MCA PI data at all four examinations in all participants (Tables 2 and 3). Irrespective of prandial state, the MCA PI values were non-significantly lower in the 2^nd^ examination compared to the 1^st^ examination. This fall in PI values (ΔPI) was larger following prolonged fasting though the difference between the two meal states was not significant (p = 0.487).

**Table 2:** Pulsatility indices in the middle cerebral artery and umbilical artery, the cerebroplacental ratio, and peak systolic velocity in the middle cerebral artery in the first and the second examination in state A and state B.

	First examination (fasting state) Mean (±SD)	Second examination Mean (±SD)	Second examination minus first examination (Δ values) Mean (±SD) (95% CI) p*	ΔB minus ΔA values Mean (±SD) (95% CI) P**
MCA-PI, State A N = 25	1.79 (±0.42)	1.60 (±0.32)	-0.19 (±0.50) (-0.39, 0.02) p = 0.071	0.07 (±0.52) (-0.14, 0.29) p = 0.487
MCA-PI, State B N = 25	1.75 (±0.29)	1.64 (±0.31)	-0.11 (±0.33) (-0.25, 0.02) p = 0.099	
UA-PI, State A N = 23	0.87 (±0.12)	0.89 (±0.10)	0.01 (±0.09) (-0.02, 0.05) p = 0.436	-0.05 (±0.12) (-0.10, 0.00) p = 0.058
UA-PI, State B N = 23	0.89 (±0.11)	0.85 (±0.09)	-0.04 (±0.09) (-0.07, 0.00) p = 0.070	
CPR, State A N = 23	2.15 (±0.62)	1.88 (±0.42)	-0.26 (±0.53) (-0.49, -0.03) p = 0.027	0.23 (±0.59) (-0.03, 0.49) p = 0.079
CPR, State B N = 23	2.01 (±0.43)	1.98 (±0.39)	-0.04 (±0.46) (-0.23, 0.16) p = 0.72	
MCA-PSV, State A N = 25	60.5 (±10.0)	60.6 (±8.1)	0.1 (±11.4) (-4.6, 4.8) p = 0.951	6.7 (±16.5) (-0.2, 13.5) p = 0.055
MCA-PSV, State B N = 25	55.7 (±8.1)	62.5 (±12.8)	6.8 (±15.3) (0.5, 13.1) p = 0.035	

*Paired t test between the values in the first and the second examination (the difference was calculated as the values at the second examination minus the values at the first examination; Δ values).

** Paired t test between the differences in ΔA (calculated as the values after prolonged fasting minus the values at the fasting state in the morning) and the ΔB values (values after SBM minus the values at the fasting state in the morning). MCA, middle cerebral artery; UA, umbilical artery; PI, pulsatility index; CPR, cerebroplacental ratio; PSV, peak systolic velocity; SBM, standard breakfast meal; A, meal state A; B, meal state B.

**Table 3:** Fetal heart rate in the middle cerebral artery and umbilical artery in the first and the second examination in state A and state B.

	First examination (fasting state) Mean (±SD)	Second examination Mean (±SD)	Second examination minus first examination (Δ values) Mean (±SD) (95% CI)	ΔB minus ΔA values Mean (±SD) (95% CI) P**
MCA-FHR, State A N = 25	136.6 (±12.0)	135.3 (±11.4)	-1.3 (±13.1) (-6.7, 4.1) p = 0.619	3.8 (±15.6) (-2.7, 10.2) p = 0.240
MCA-FHR, State B N = 25	136.2 (±12.7)	138.6 (±8.6)	2.4 (±11.2) (-2.2, 7.1) p = 0.286	
UA-FHR, State A N = 23	139.2 (±11.3)	135.7 (±10.7)	-3.5 (±9.8) (-7.8, 0.7) p = 0.099	1.7 (±17.3) (-5.8, 9.2) p = 0.635
UA-FHR, State B N = 23	140.7 (±11.0)	138.9 (±8.8)	-1.8 (±13.3) (-7.6, 4.0) p = 0.528	

*Paired t test between the values in the first and the second examination (the difference was calculated as the values at the second examination minus the values at the first examination; Δ values).

** Paired t test between the differences in ΔA (calculated as the values after prolonged fasting minus the values at the fasting state in the morning) and the ΔB values (values after SBM minus the values at the fasting state in the morning). MCA, middle cerebral artery; UA, umbilical artery; PI, pulsatility index; FHR, fetal heart rate; SBM, standard breakfast meal; A, meal state A; B, meal state B.

Complete UA PI data were obtained in 23 participants (Tables 2 and 3). Although the UA PI values in the 2^nd^ examination were lower than the 1^st^ examination for state B, and higher for state A, none reached statistical significance. The difference between the two prandial states (ΔPI), was also not significant (p = 0.058). There were no significant differences observed in the baseline values of MCA PI and UA PI between day 1 and day 2 (Table 4).

**Table 4:** MCA-PI, UA-PI, FHR, CPR, and MCA-PSV values in the first examination study day 1 and study day 2 (baseline values).

	1^st^ examination study day 1 Mean (±SD)	1^st^ examination study day 2 Mean (±SD)	Change Mean (±SD) (day 2 minus day 1) (95% CI)	p*
MCA-PI N = 25	1.82 (±0.27)	1.72 (± 0.43)	0.10 (±0.44) (-0.09, 0.28)	0.290
UA-PI N = 23	0.87 (±0.10)	0.89 (±0.13)	-0.02 (±0.12) (-0.07, 0.04)	0.549
CPR N = 23	2.13 (±0.45)	2.03 (±0.60)	0.11 (±0.58) (-0.14, 0.36)	0.382
MCA-FHR (beats/min) N = 25	136.8 (±12.1)	136.0 (±12.5)	-0.80 (±13.5) (-6.4, 4.8)	0.769
UA-FHR (beats/min) N = 23	140.2 (±12.0)	139.8 (±10.4)	0.4 (±13.2) (-5.3, 6.2)	0.876
MCA-PSV (cm/sec) N = 25	57.3 (±7.1)	58.8 (±11.2)	-1.5 (±11.9) (-6.4, 3.4)	0.526

*Paired t test between the values in the first examination (fasting state morning) study day 1 and study day 2. MCA, middle cerebral artery; UA, umbilical artery; PI, pulsatility index; CPR, cerebroplacental ratio; PSV, peak systolic velocity; FHR, fetal heart rate.

### Δ PI adjusted for ΔFHR

There were no significant differences observed in FHR between 2^nd^ and 1^st^ examination in meal state A and B, in the baseline FHRs, and in ΔFHR between the prandial states in either MCA or UA.

In the MCA, ΔPI was negatively correlated with ΔFHR. This correlation was statistically significant in state A (r = -0.46, p = 0.022), but not in state B (r = -0.31, p = 0.126).

In the UA, the correlation between ΔPI and ΔFHR was negative and significant in state B (r = -0.43, p = 0.041), and positive but not significant in state A (r = 0.32, p = 0.141) (S1 Table).

In the linear mixed model analysis (with adjustment for the influence of ΔFHR) the difference in the ΔMCA PI and ΔUA PI were not significant between state A and state B (p = 0.179, p = 0.064, respectively).

### CPR

CPR was calculated using the PI data, unadjusted for FHR (n = 23) (Table 2). Compared to the fasting state examination in the morning, CPR was significantly lower following the prolonged fast, with no significant difference in CPR observed post-prandially. There was no significant difference in the ΔCPR values between state A and B or in the baseline CPR values (Table 4).

### MCA-PSV

The MCA-PSV values increased significantly after food intake, whereas no significant increase was observed following the prolonged fast. There was no significant difference in the ΔPSV between the two meal states or in the baseline values of MCA-PSV (Tables 2 and 4).

## Discussion

Our main hypothesis of a decrease in the vascular resistance of the fetal cerebral circulation following a standard breakfast meal compared to prolonged fasting was rejected. The MCA PI and UA PI values, adjusted and unadjusted for FHR, were not significantly different between the two meal states. Irrespective of meal state, MCA PI values unadjusted for FHR were non-significantly lower at midday (second examination). We observed no significant differences in FHR, CPR or MCA-PSV dependent on meal state.

Our findings are in line with the only previous crossover study by Yasuhi et al.[11]. Regardless of the maternal meal state, no significant change was observed in MCA PI. However, Yasuhi et al. did not adjust the MCA PI values for FHR. In this study, we have adjusted for the potential influence of FHR on the MCA-PI values. Our results indicate diurnal variations in the MCA PI. Opheim et al. demonstrated a significant postprandial decrease in MCA PI [17], but the study did not include a control group not exposed to food, and an influence of diurnal variations could not be ruled out.

A few larger studies have examined the effect of extended overnight fast on MCA Doppler indices though none adjusted their measurements for FHR. In a study with 110 healthy women with 10–12 h extended overnight fast at mean GW 34 (range; 28–39 weeks), Abd-El-Aal et al. observed no significant differences in MCA PI values from before (10:00 am-12:00 pm) to 2 hours post-prandially (1:00–3:00 pm) [28]. In the largest case-control study with 210 fasting women and 240 non-fasting women in late third trimester, Abd-Allah et al. did not find any impact of maternal fasting (12–16 h) on fetal MCA PI values [29]. Women in both groups were examined in the afternoon at 3:00–6:00 pm. Although the case-control measurements were performed later during the day, the results of the two studies are comparable. Mean MCA PI values post-prandially and after extended fast in the present study are in line with values obtained at 7:00 and 10:00 am in the crossover study by Yasuhi et al. [11]. However, the current values were profoundly lower than in the study by Abd-Allah et al., which was performed in the evening (1.64 and 1.60 vs. 2.21 and 2.22, respectively) [29]. These findings may reflect fluctuations in diurnal variations in the MCA PI values.

The current study is the first crossover study that has explored UA PI adjusted for FHR between two different maternal meal states. The UA PI values, both unadjusted and adjusted for FHR, did not identify significant differences between the two maternal meal states, indicating the presence of diurnal variations. This study confirms the findings of other investigators who have not been able to demonstrate a significant difference in UA vascular resistance indices in various pre- and post-prandial states: extended fasting [11, 28, 29], glucose drink versus a water drink [30], before and after a maternal meal [17], or before and after an oral glucose tolerance test [16, 31]. In contrast, Degani et al. reported an increase in unadjusted UA PI values following an oral glucose tolerance test [32].

The present study is the first blinded, crossover study evaluating the impact of maternal meal state on CPR. The results show no significant difference between the two maternal meal states. The results denotes diurnal variations in the CPR. These results are in agreement with evaluation of CPR in two different case-control studies, with women in an extended fast versus non-fasting state during the third trimester [29, 33]. In contrast, Opheim et al. demonstrated a significant post-prandial fall in CPR values, but could not control for diurnal variations [17].

To our knowledge, this is also the first study with a crossover design examining fetal MCA-PSV in two different maternal meal states. Following the SBM (meal state B) we observed a significant increase in the MCA-PSV values. However, this increase was not significant (p = 0.055) when compared with prolonged fasting (meal state A), indicating that MCA-PSV might be influenced by diurnal variations independent of maternal food intake. The mean increase in MCA-PSV following SBM was of the same magnitude in the present study as in our previous study (6.8 versus 5.9 cm/sec). These values are comparable to those obtained by Avitan et al. showing an increase in MCA-PSV from an examination in the morning (8:00 am) to another examination in the afternoon (1:30 pm). Both examinations in that study were preceded by a meal. The differences between the two examinations in the study by Avitan et al. could thus represent diurnal variations [34].

Fetal breathing movements and behavioral state may influence Doppler blood flow velocity waveforms [13, 30, 35]. In the present study, all velocity waveforms were recorded with fetuses in the most inactive state possible; there were no significant differences in fetal body movements dependent on maternal meal state.

There are limitations to our study. Initially, we planned the study as a randomized controlled trial. Due to convenience issues, most of the women received the SBM the first examination day. We found no significant differences between the women who received SBM on study day 1 versus study day 2 for the following variables: maternal age, gestational age at examination or at delivery, fetal gender, neonatal weight or morning glucose values. The order of the meal state was blinded for the ultrasound examiner and for those involved in the statistical analyses. In a subgroup analysis of the MCA PI and glucose values based on the order of meal state, the findings were in line with the results reported for the total group (S2 Table). We find it unlikely that the treatment the first day (prolonged fasting or receiving the SBM) would affect the results of the opposite treatment 1 to 3 days later. Other limitations of this study are the lack of simultaneous cardiotocography recordings, duration of sleep and a standardized classification and measurement of fetal behavioral state. The sample size was calculated to evaluate MCA PI values. Thus, it is not certain whether the study was of sufficient size to draw valid conclusions for the other measured variables. Our power calculation was based on a fall in MCA-PI following a maternal meal and the assumption of no change following extended fasting.

The strength of the study resides in being the largest blinded crossover study performed by a single examiner with estimation of MCA PI and UA PI with adjustment for FHR, and the only crossover study to evaluate MCA-PSV and CPR in a narrow gestational age range.

The study included low risk pregnancies to establish the physiological effect of a maternal meal on fetal cerebrovascular resistance. Fetuses with growth restriction are more vulnerable to the “brain-sparing” effect. Further exploration is necessary to investigate if this clinically important subgroup of fetuses possesses a different physiological pattern and a different response to maternal food intake.

## Conclusion

In uncomplicated pregnancies our results show diurnal variations in MCA PI whereas maternal food intake does not have any significant impact on these values. Performing follow-up examinations at the same time of the day will reduce the effect of diurnal variations in the measurements.

## Supporting information

S1 Table Correlation between fetal Doppler blood flow variables and fetal heart rate in state A and B. (PDF)

S2 Table Based on the order of meal state, an analysis of the MCA-PI values between state A and state B and glucose values between study day 1 and 2. (PDF)
