# Supplementary material for: Effect of a standardized maternal meal on fetal middle cerebral artery Doppler indices: A single-blinded crossover study
Source: PLoS One. 2022 Aug 4;17(8):e0272062. doi: 10.1371/journal.pone.0272062 (PMC9352093; doi:10.1371/journal.pone.0272062)
Supplement: S1 Table — (PDF) [file pone.0272062.s001.pdf]

Table S1. Correlation between fetal Doppler blood flow variables and fetal heart rate in state A and B.

	State A			State B		
	FHR in first examination	FHR in second examination	Change in (Δ) FHR	FHR in first examination	FHR in second examination	Change in (Δ)FHR
MCA-PI in first examination	r = -0.25<br>p = 0.225			r = -0.45<br>p = 0.025		
MCA-PI in second examination		r = -0.41<br>p = 0.042			r = -0.06<br>p = 0.770	
Change in (Δ) MCA-PI			r =-0.46<br>p = 0.022			r = -0.31<br>p = 0.126
UA-PI in first examination	r = -0.18<br>p = 0.423			r = -0.20<br>p = 0.359		
UA-PI in second examination		r = -0.29<br>p = 0.187			r = -0.06<br>p = 0.802	
Change in (Δ) UA-PI			r = 0.32<br>p = 0.141			r = -0.43<br>p = 0.041

Changes in the different variables (Δ) are calculated as the values in the second examination minus the values in the first examination.
r = Pearson’s correlation coefficient. MCA, middle cerebral artery; PI, pulsatility index; FHR, fetal heart rate; State A, values in the fasting state in the morning and prolonged fasting; State B, values in the fasting state in the morning and values after SBM; SBM, standard breakfast meal.
