# Supplementary material for: Effect of a standardized maternal meal on fetal middle cerebral artery Doppler indices: A single-blinded crossover study
Source: PLoS One. 2022 Aug 4;17(8):e0272062. doi: 10.1371/journal.pone.0272062 (PMC9352093; doi:10.1371/journal.pone.0272062)
Supplement: S2 Table — (PDF) [file pone.0272062.s002.pdf]

Table S2. Based on the order of meal state, an analysis of the MCA-PI values between state A and state B and glucose values between study day 1 and 2.

	Change second examination minus first examination in State A (ΔA values) Mean (±SD) (95% CI)	Change second examination minus first examination in State B (ΔB values) Mean (±SD) (95% CI)	Difference in ΔB minus ΔA values Change Mean (±SD) (95% CI) *p-value	Study day 1 Mean (±SD) (95% CI)	Study day 2 Mean (±SD) (95% CI)	Difference in day 1 minus day 2 values Mean (±SD) (95% CI) **p-value
MCA-PI, Meal order AB N=7	-0.34 (±0.31) (0.63, -0.05)	-0.21 (±0.38) (-0.56, 0.14)	0.13 (±0.44) (-0.27, 0.53) p=0.454			
MCA-PI, Meal order BA N=18	-0.13 (±0.54) (-0.40, 0.14)	-0.08 (±0.31) (-0.23, 0.08)	0.05 (±0.56) (-0.23, 0.33) p=0.702			
Glucose level in fasting morning state (mmol/L), Meal order AB N=7				4.40 (±0.30) (4.12, 4.68)	4.59 (±0.33) (4.28, 4.89)	-0.19 (±0.43) (-0.59, 0.22) p=0.300
Glucose level in fasting morning state (mmol/L), Meal order BA N=17				4.31 (±0.27) (4.17, 4.47)	4.31 (±0.32) (4.14, 4.47)	0.05 (±0.56) (-0.23, 0.33) p=0.702

*Paired t test between the differences in ΔA (calculated as the values after prolonged fasting minus the values at the fasting state in the morning) and the ΔB values (values after SBM minus the values at the fasting state in the morning).

**Paired t test between the differences in study day 1 and 2.

MCA, middle cerebral artery; PI, pulsatility index; A, meal state A; B, meal state B; SBM, standard breakfast meal.
